# The impact of informal social support on the health poverty vulnerability of the elderly in rural China: based on 2018 CHARLS data

**DOI:** 10.1186/s12913-022-08468-3

**Published:** 2022-09-05

**Authors:** Gaoling Wang, Xiaolin Shen, Zhaopeng Cheng, Qianqian Kan, Shaoliang Tang

**Affiliations:** School of Health Economics and Management, Nanjing University of Chinese Medicine, Nanjing, China

**Keywords:** Rural elderly population, Health poverty vulnerability, Informal social support

## Abstract

**Objective:**

From the perspective of informal social support, this paper analysed the impact of factors such as “Relationship with spouse”, “Relationship with Children”, “Financial support from children”, “Sibling support”, “Support from other friends and relatives” and “Borrowing costs” on the health poverty vulnerability of elderly people in rural China.

**Methods:**

Based on the data of the China Health and Retirement Longitudinal Study (CHARLS) in 2018, the vulnerability of the rural elderly to health poverty was measured from two dimensions of health status and influencing factors of health status by the three-stage feasible generalized least square method. A quantile regression model was used to analyse the impact of six variables in the informal social support network on health poverty vulnerability: “Relationship with spouse”, “Relationship with children”, “Financial support from children”, " Sibling support”, " Support from other friends and relatives”, and “Borrowing costs”.

**Results:**

When the poverty line standards were 2995 CNY/year and 4589 CNY/year, the health poverty vulnerability of the elderly population in rural China was 0.397 and 0.598 in 2018. In the analysis of informal social support, factors such as the relationship with spouse, relationship with children, borrowing costs, support from other friends and relatives, and sibling support had different impacts on the health poverty vulnerability of the rural elderly, who were classified into three groups according to their different vulnerabilities.

**Conclusion:**

According to the analysis of the 2018 CHARLS database, the health poverty vulnerability of the elderly population was related to the informal social support network, and it is necessary to pay attention to the role of informal channels such as children, spouses, relatives and friends in daily care and financial support for rural elderly individuals. Meanwhile, the government and other formal organizations should also give full play to their supporting role for elderly individuals, who are highly vulnerable to health poverty, and their families.

## Introduction

There is no end to poverty governance, and the issue of poverty needs to be widely considered by all sectors of society. Early warning and intervention of returning to poverty due to illness is an important work of health poverty control. After 2020, the targets of health poverty management in China shifted to rural key groups, such as disabled people, chronic disease patients and elderly people without families [1]. Elderly individuals in rural areas are more likely to fall into poverty due to physiological degradation, reduced ability to adapt, and weakened family support function [2]. The elderly population in rural areas has become a key group in health poverty management. How can health poverty risk be monitored? What intervention measures should be taken? This study will take this as the research background.

In 2001, the World Bank [3] proposed the concept of poverty vulnerability, which is a prospective indicator to measure the possibility of poverty. With the enrichment and improvement of Micro-data, an increasing number of scholars have begun to find empirical support for the theoretical mechanism of poverty vulnerability. For example, Celidoni M [4] used micro data to decompose and measure the vulnerability of individuals to poverty. Sohns F [5] used survey data to empirically study the impact of entrepreneurship on family poverty vulnerability. Jinguang Guo used survey data to measure and analysed the vulnerability and poverty dynamics of poor people in China [6]. Yue Liu et al. [7] proposed the concept of “vulnerability to health poverty” and defined it as the possibility of family or individual welfare falling below the poverty line due to the impact of health-related risks, which reflects the probability of falling into poverty in the future. In the poverty alleviation era, inhibiting the return to poverty due to illness has become the main target of the health of poor governance, and health poverty vulnerability can be used as a proactive monitoring of early warning indicators used in health poverty governance to explore health-related risk factors in the elderly population in the future and the probability of falling into poverty. It will transform postengagement measures of health poverty into prior measures [8]. The concept of health poverty vulnerability has appeared in the field of health poverty risk monitoring and research, but no standard evaluation index has been established.

How to reduce vulnerability to health poverty has been discussed by previous scholars from the aspects of policy, social environment and social support networks. Yan Wei conducted an empirical analysis of rural women’s health poverty vulnerability based on the special survey data of “Targeted health poverty Alleviation and Population Development” in five provinces of China and explored its influencing factors by using the Tobit model [9]. Yue Liu used the word frequency analysis method to explore the risk factors and evaluation index system of health poverty vulnerability of rural families and found that health poverty vulnerability is not only affected by the internal characteristics of the family but also has a certain connection with the external environment of the family [10]. Based on existing studies, this paper selects the research perspective of informal support in social support to discuss and analyse the influencing factors of the health poverty vulnerability of the elderly in China. Jieyi Hu believed that social support networks can help poor people resist risks and improve their ability to fend off the risk [11]. Social support is mainly divided into formal support and informal social support. Cullen M believed that social support is the material and spiritual help that individuals receive from communities and social networks [12], which includes formal organizations and nonorganizations. Informal social support, also known as unofficial social support and informal care [13], is different from formal support from the official organization. Informal social support mainly refers to the social support network based on geographical and kinship relations form, which includes financial and material help as well as emotional, behavioral and informational support from family members, friends and neighbors [14–16]. As an important part of the social support network, informal social support plays an important role in reducing the risk of falling into poverty and returning to poverty. Due to the limited conditions in rural areas, formal support has little impact, and informal social support is the main source of social support for rural residents, especially parents, children and spouses [17]. The impact of informal social support on health is an important part of the research on the health effects of social support. Studies show that informal social support from family and friends can relieve pressure and promote physical health. People with a higher level of informal social support have lower social pressure and a higher health level [18]. Krause N found that informal social support has a positive impact on the health self-assessment of the elderly [19]. Bonsang E, found in his research that the geographical distance of children has a potential impact on informal care [20]. Nocon A and Pearson M found that friends and neighbors are important parts of the elderly support system [13]. According to Adams R, when elderly individuals live alone, they are likely to rely on friends and neighbors to establish similar family relationships [21]. Seeman and Berkman believed that those elderly without spouses rely on family members as instrumental support sources, while friends rely on emotional support sources [22]. However, according to Adams R, the support provided by friends and neighbours is limited to practical help such as work and transportation, and they rarely provide close care [21]. Researches by Chinese scholars on informal social support have mainly focused on the impact of informal social support on family poverty status and poverty governance. Fang Han proposed that the lack of an informal social support social network is not conducive to reducing the economic burden of poor families and will lead to the loss of more opportunities for them to obtain help [23]. Jieyi Hu et al. believed that informal social support networks can help individuals and families enhance their ability to cope with risks and reduce their vulnerability to poverty [11]. The study of Xiaoliang Hong et al. [24] found that support among relatives and friends is an important factor affecting health poverty, among which kinship plays a leading role in the family support network, and nonrelatives also have great support in employment.

According to the literature, scholars have mainly studied the poverty vulnerability of the elderly population from the perspective of social support and studied its impact on the health level of the population and the poverty state of the family from the perspective of informal support. Further research is needed on the impact of informal social support on the health poverty vulnerability of the rural elderly population. Therefore, this study proposes relevant hypotheses: First, under the current social environment, does informal social support have an impact on the health poverty vulnerability of the elderly population in rural China? Are there any differences in the impact of informal social support on the health poverty vulnerability of elderly individuals in rural China among different subjects, such as children, spouses, and relatives?

In 2018, the poverty line in China’s rural areas was raised to 2,995 CNY per year based on the China Rural Poverty Monitoring Report. According to the standard of the World Bank, the international poverty line was $1.90 per person per day. According to the Statistical Bulletin of National Economic and Social Development for 2018, the average exchange rate of CNY for the year was 1 USD to 6.6174 CNY. Based on the exchange rate conversion for that year, the international poverty line expressed in this paper is 4589 CNY/year (1.90*6.6174*365). This study selected the data of the China Health and Retirement Longitudinal Study (CHARLS) in 2018, took informal social support as the research entry point, and took the support among relatives and friends as the main research perspective. A quantile regression model was used based on the poverty lines of 2995 CNY/year and 4589 CNY/year to study the impact mechanism of informal social support on the health poverty vulnerability of elderly people in rural China.

## Methods

### Data sources

Using CHARLS as a data source, this study evaluated the vulnerability of the rural elderly population to health poverty. The China Health and Retirement Longitudinal Study aims to collect a nationally representative sample of Chinese residents more than 45 years old, designed to serve the scientific research needs relating to older people. The National Baseline field survey was launched in 2011 and included approximately 10,000 households and 17,500 people in 150 districts and 450 village/residential councils who were followed up every two years, with all data made public one year after the end of data collection [25]. The CHARLS can provide a wealth of information when studying the health and pension issues of the elderly population in China, including basic information of respondents, health status, cognition and depression, medical care and insurance, work and retirement [26]. The CHARLS database has good reliability and validity and can better reflect the actual situation of the elderly population in China [25].

**Fig. 1:**
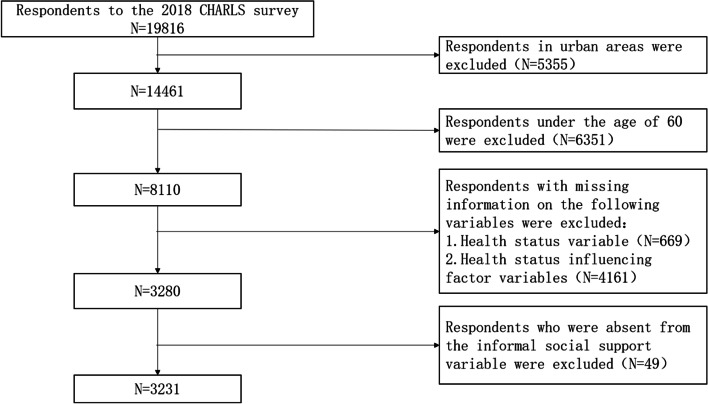
Respondents’ flow in the study

CHARLS 2018 collected information from 19,816 respondents. As the research object of this paper is the elderly in rural areas, 5,355 interviewees in urban areas and 6,351 interviewees under the age of 60 are excluded. Of the remaining respondents, 669 lacked information on variables of their personal health status, and 4,161 lacked information on variables of factors influencing their health status. In addition, this study excluded 49 interviewees who lacked information on informal social support variables and finally included 3231 rural elderly people. The sample selection process is shown in Fig. 1. As CHARLS has been approved by the Ethics Committee of Peking University and is open to the whole community, no additional ethical approval is needed.

### Independent variable

As a whole, informal social support has multiple functions, including economic support, emotional support and life care [27]. According to hierarchical supplementary theory, elderly care is sorted according to the principle of close relatives: spouses, adult children and distant relatives [28]. Xiaotong Fei proposed the difference order theory that informal social support in Chinese society is ordered sequentially, beginning with close relatives and then distant relatives, friends, and finally neighbors [29]. Morinaga and Tomomi believed that the comprehensive model of informal social support networks should include the scale of the network, density of network relationships, degree of connection, strength and persistence of network relationships, guidance and reciprocity, and degree of help [30]. Gang Li believed that kinship plays a greater role in supporting poor families than nonkinship, and relatives can provide economic and labor support for families. In terms of nonkinship, neighbors and friends can also provide employment support for members of poor families [31]. The above literature shows that children, spouses, relatives and friends are the main forces in informal social support. Therefore, this paper combined the actual situation of the rural elderly population in China and the characteristics of database indicators from the informal social support network density and degree of contact and selected “Relationship with spouse” and “Relationship with Children”. Starting from the scale of network relationships, four indicators were selected, including “Borrowing costs”, “Sibling support”, “Support from other friends and relatives”, and “Financial support from children” (Borrowing costs included the total amount of loans outstanding, excluding mortgages and credit cards;Credit card balance;The amount owed to other families, individuals and units, excluding loans and credit card arrears) was used to establish an evaluation indicator system for vulnerability to health poverty in informal social support networks, and this system was used as the main independent variable in quantile regression analysis (see Table 1).

**Table 1:** Selection and assignment of informal social support indicators

First level indicators	Indicator description and assignment	Variable code
Relationship with spouse	1=“Extremely satisfied”;2=“Greatly satisfied”;3=“Satisfied”;4=“Less satisfied”;5=“Completely dissatisfied”	X_1_
Relationship with Children	1=“Extremely satisfied”;2=“Greatly satisfied”;3=“Satisfied”;4=“Less satisfied”;5=“Completely dissatisfied”	X_2_
Financial support from children	Numerical variable	X_3_
Sibling support	Numerical variable	X_4_
Support from other friends and relatives	Numerical variable	X_5_
Borrowing costs	Numerical variable	X_6_

### Dependent variable

Poverty measurement is an indispensable way to study poverty. The key step of health poverty elimination is how to measure the vulnerability of poverty. According to the previous literature review, the vulnerability of poverty measurement can be divided into three ways: (1) Vulnerability as expected poverty, VEP. (2) Vulnerability as Low Expected Utility, VEU (3) Vulnerability as Uninsured Exposure to Risk, VER. Among the three measures, VER belongs to postmortem analysis, lacks foresight and has certain limitations in practical application. However, the household utility function involved in the VEU measure is difficult to calculate accurately according to the existing data, and the estimated results also have a certain deviation. In contrast, the VEP data are more convenient to obtain and are a kind of advanced estimation of future poverty, which is the easiest to realize and the most commonly used measurement method at present [32]. The VEP measure was adopted in this study, and the measured health poverty vulnerability was taken as the dependent variable.

### Health poverty vulnerability measurement indicators

To measure the vulnerability of the elderly to health poverty in rural China, we needs to consider the impact of various factors on the health poverty of elderly individuals. Therefore, this paper explored the research results of relevant scholars, investigated the related risk factors that can cause health poverty and established a measurement indicator system. Xiaolei Yang’s empirical analysis found that the living conditions of poor elderly people are related to their age, marriage status, health status and pension insurance payment [33]. Changyong Yu found that chronic diseases have a significant impact on rural elderly poverty, and rural elderly poverty has obvious heterogeneity [34]. Disease risk theory emphasizes the management of the whole process and life cycle of health. Beilei Yan pointed out that in the process of disease risk, health care and healthy living conditions can be used to analyse the disease risks affecting patients with chronic diseases [35]. Hua Han constructed a mechanism framework for the formation of absolute poverty among the elderly in rural areas from the perspective of material deprivation, indicating that medical treatment level is an important factor for poverty among the elderly in rural areas [36]. Yan Wei believed that disease prevention can effectively improve one’s ability to resist risk shocks and reduce the probability of poverty in the future. Meanwhile, she pointed out that the lack of access to medical services in rural areas and the limited professional level of rural doctors have increased the health risks of residents [9]. This paper measured the vulnerability of the rural elderly to health poverty from the two aspects of health status and health status influencing factors by combining the characteristics of the rural elderly population and the current situation of the health service system in China (see Table 2).

**Table 2:** Selection and assignment of health poverty vulnerability measurement indicators

First level indicators	The secondary indicators	Indicator description and assignment	Variable code
Health status of the elderly	Self-evaluation of health	Very healthy = 1, Healthier = 2, Healthy = 3, Unhealthy = 4	a_1_
	Pain	Yes = 1, No = 0	a_2_
	Chronic disease	Yes = 1, No = 0	a_3_
	Gender	Male = 1, Female = 2	a_4_
	Age	Numerical variable	a_5_
	Marital status	Married (living with spouse) = 1, Separation/divorce = 2, Widowed/unmarried = 3	a_6_
Factors influencing the health status of the elderly	Participating in the new rural cooperative medical insurance	Yes = 1, No = 0	a_7_
	Taking out critical illness insurance	Yes = 1, No = 0	a_8_
	Whether medicaid	Yes = 1, No = 0	a_9_
	Received medicaid last year	Numerical variable	a_10_
	Master self-treatment	Yes = 1, No = 0	a_11_
	Smoking	Yes = 1, No = 0	a_12_
	Drinking	Yes = 1, No = 0	a_13_
	Checking blood pressure regularly	Yes = 1, No = 0	a_14_
	Checking for diabetes often	Yes = 1, No = 0	a_15_
	Medical distance	Numerical variable	a_16_
	Medical service satisfaction	1=“Extremely satisfied”;2=“Greatly satisfied”; 3=“Satisfied”; 4=“Less satisfied”; 5=“Completely dissatisfied”	a_17_
	Number of hospitalizations in a year	Numerical variable	a_18_
The annual income of the elderly	The income received by individuals in the past year(excluding pension)	Numerical variable	b_1_
	The pension that an individual receives	Numerical variable	b_2_

### Health poverty vulnerability measurement model

In this study, heteroscedasticity White’s test was performed on the data. According to the test results, P = 0.000 < 0.05 indicates that the model has heteroscedasticity. Therefore, the ordinary least squares method used to estimate the review will not be an effective estimation of the model parameters and the best model. Therefore, correction for heteroscedasticity is necessary. Integrating various heteroscedasticity in the end, this research chose the three-stage feasible generalized least squares method (FGLS) for the heteroscedasticity regression model of correction. FGLS is a common method to eliminate heteroscedasticity. Its advantage lies in that first-order autoregression and cross-sectional heteroscedasticity are allowed in the panel during the estimation process without affecting the accuracy of the estimation results [37].

The specific operation process of this paper is as follows:

- Step 1: Estimate the income equation and residual equation of the rural elderly population. The annual income $Ln{Y}_{h}$of the elderly is selected as the explained variable, and the explanatory variable ${X}_{h}$ is extracted from two dimensions,includ, ng Health status of the elderly (Self-evaluation of health, Pain, Chronic disease)and Factors influencing the health status of the elderly (Gender, Age, Marital status, Participating in the new rural cooperative medical insurance, Taking out critical illness insurance, Whether Medicaid, Received medicaid last year, Master self-treatment, Smoking, Drinking, Checking blood pressure regularly, Checking for diabetes often, Medical distance, Medical service satisfaction, Number of hospitalizations in a year).

1 $$Ln{Y}_{h}={X}_{h}{\beta }_{h}+{e}_{h}$$

2 $${\widehat{{e}_{h,ols}}}^{2}={X}_{h}\theta +{\varpi }_{h}$$

- Step 2: Estimate the expected value and variance of the logarithmic annual income of the rural elderly population. Based on the first step, ${x}_{h}\widehat{{\theta }_{ols}}$ was used as the weight to make weighted FGLS regression to receive log income expectation $\widehat{E}\left(Ln{Y}_{h}|{X}_{h}\right)$ and variance $\widehat{V}\left(Ln{Y}_{h}|{X}_{h}\right)$ of rural elderly.

3 $$\widehat{E}\left(Ln{Y}_{h}|{X}_{h}\right)={X}_{h}{\widehat{\beta }}_{FGLS}$$

4 $$\widehat{V}\left(Ln{Y}_{h}|{X}_{h}\right)={\widehat{\sigma }}_{e,h}^{2}={X}_{h}{\widehat{\theta }}_{FGLS}$$

- Step 3: Calculate the health poverty vulnerability of elderly individuals. This paper assumes that the income level of the rural elderly follows a normal distribution. $Lnz$ is the log of the poverty line. The calculation method is shown in Formula (5). This paper calculates the health poverty vulnerability of the rural elderly based on the poverty lines of 2995 CNY/year and 4589 CNY/year.

5 $$\widehat{{PV}_{h}}=\widehat{P}\left(Ln{Y}_{h}\le Lnz|{X}_{h}\right)={\varnothing }\frac{(Lnz-{X}_{h}\widehat{\beta })}{\sqrt{{X}_{h}\widehat{\theta }}}$$

### Quantile regression

This study adopted the method of quantile regression and takes the health poverty vulnerability of the elderly population as the dependent variable. To explore whether six independent variables, including “Relationship with spouse (X1)”, “Relationship with children (X2)”, " Financial support from children (X3)”, " Sibling support (X4)”, " Support from other friends and relatives (X5)” and “Borrowing costs (X6)”, have a significant impact on the health poverty vulnerability and the degree of impact. Quantile regression is an extension of the ordinary least square method. Ordinary least square regression describes the influence of independent variables on the mean value of dependent variables, while quantile regression regresses independent variables according to the conditional quantile of dependent variables, so it can give a more comprehensive and accurate description of the conditional distribution of dependent variables. Meanwhile, the quantile regression method can describe the tail features of the data distribution, and the coefficient is more robust than the ordinary least square regression, as shown in formula (6) for the calculation method.

6 $${\mathcal{Q}}_{y}\left(\gamma ,x\right)={a}_{0}+{a}_{1}{x}_{1}+\dots +{a}_{k}{x}_{k}+{Q}_{n}\left(\gamma \right)$$

## Results

### Sample characteristics results

This study provides descriptive statistical results on sample characteristics, as detailed in Tables 3 and 4.

**Table 3:** Descriptive statistics of health poverty vulnerability measurement samples

Ordered variable	Value	Freq	%	Numerical variable	mean	std
Self-evaluation of health	1(Very healthy)	348	10.77	Age	68.442	6.473
	2(Healthier)	303	9.38	Received medicaid last year	14.1272	400.849
	3(Healthy)	1410	43.64	Medical distance	11.6591	18.7078
	4(Unhealthy)	1170	36.21	Number of hospitalizations in a year	0.3423	0.8793
Pain	1(Yes)	2131	65.95			
	0(No)	1100	34.05			
Chronic disease	1(Yes)	1573	48.68			
	0(No)	1658	51.32			
Gender	1(Male)	1567	48.50			
	2(Female)	1664	51.50			
Marital status	1(living with spouse)	2608	80.72			
	2(Separation/divorce)	33	1.02			
	3(Widowed/unmarried)	590	18.26			
Participating in the new rural cooperative medical insurance	1(Yes)	2928	90.62			
	0(No)	303	9.38			
Taking out critical illness insurance	1(Yes)	116	3.59			
	0(No)	3115	96.41			
Whether medicaid	1(Yes)	13	0.40			
	0(No)	3218	99.60			
Master self-treatment	1(Yes)	2031	62.86			
	0(No)	1200	37.14			
Smoking	1(Yes)	90	2.79			
	0(No)	3141	97.21			
Drinking	1(Yes)	962	29.77			
	0(No)	2269	70.23			
Checking blood pressure regularly	1(Yes)	1270	39.31			
	0(No)	1961	60.69			
Checking for diabetes often	1(Yes)	106	3.28			
	0(No)	3125	96.72			
Medical service satisfaction	1(Extremely satisfied)	571	17.67			
	2(Greatly satisfied)	797	24.67			
	3(Satisfied)	1334	41.29			
	4(Less satisfied)	215	6.65			
	5(Completely dissatisfied)	314	9.72			

**Table 4:** Descriptive statistics of sample influencing factors of informal social support

Ordered variable	Value	Freq	%	Numerical variable	mean	std
Relationship with spouse	1(Extremely satisfied)	160	4.95	Financial support from children	3093.56	9737.06
	2(Greatly satisfied)	1118	34.60	Sibling support	1.7951	33.89
	3(Satisfied)	1254	38.81	Support from other friends and relatives	507.397	3825.04
	4(Less satisfied)	168	5.20	Borrowing costs	4126.06	29464.6
	5(Completely dissatisfied)	531	16.43			
Relationship with Children	1(Extremely satisfied)	245	7.58			
	2(Greatly satisfied)	1609	49.80			
	3(Satisfied)	1217	37.67			
	4(Less satisfied)	114	3.53			
	5(Completely dissatisfied)	46	1.42			

### FGLS regression analysis of the health poverty vulnerability of the rural elderly population

In this paper, the annual income of the elderly was defined as the total annual actual income of individuals, including the income received by individuals in the past year, excluding pension;the pension that an individual receives. In this paper, FGLS regression analysis was performed on the sample data by STATA 14.0 software, and the estimated results are shown in Table 5. When the explained variable was the logarithm of the annual income of the rural elderly population, it can be found that at the significance level of 5%, self-assessment of health, pain, gender, age, checking blood pressure regularly, checking for diabetes often, participating in the new rural cooperative medical insurance, mastering self-treatment and received medicaid last year were significant. When the explained variable was the logarithm of the square of the residual term, it was found that at the significance level of 5%, whether medicaid, received medicaid last year, gender, age and other indicators were significant, which were the main factors affecting the logarithm function of their income, as well as the main factors affecting their annual income.

**Table 5:** FGLS estimation results of the rural elderly annual income model

Explanatory variables	Explained variable: logarithm of annual income of rural population		Explained variable: the logarithm of the squared residual term	
	Coefficient estimation	T value	Coefficient estimation	T value
Self-evaluation of health	-0.032^a^ (0.011)	-2.80	-0.478^a^	-10.22
			(0.047)	
Pain	-0.071^a^ (0.022)	-3.29	-0.377^a^	-4.24
			(0.089)	
Chronic disease	-0.004	-0.28	-0.417^a^	-5.02
	(0.016)		(0.083)	
Gender	-0.124^a^	-4.13	-2.311^a^	-26.19
	(0.030)		(0.088)	
Age	-0.004^a^	-4.07	-0.038^a^	-5.99
	(0.001)		(0.006)	
Marital status	-0.007	-0.78	0.096	1.81
	(0.009)		(0.053)	
Smoking	0.036	0.58	0.292	1.25
	(0.062)		(0.234)	
Drinking	0.031	1.22	0.421^a^	4.51
	(0.025)		(0.093)	
Checking blood pressure regularly	0.033^a^	2.30	0.110	1.33
	(0.015)		(0.083)	
Checking for diabetes often	0.123^a^	2.80	0.612^a^	2.81
	(0.044)		(0.218)	
Participating in the new rural cooperative medical insurance	-0.484^a^	-4.27	-2.892^a^	-21.64
	(0.113)		(0.134)	
Taking out critical illness insurance	0.005	0.10	0.638^a^	3.08
	(0.047)		(0.207)	
Whether medicaid	-0.173	-0.49	1.368^a^	2.23
	(0.353)		(0.613)	
Master self-treatment	-0.053^a^	-3.11	-0.400^a^	-4.81
	(0.017)		(0.083)	
Medical distance	0.001	1.48	0.008^a^	3.69
	(0.0001)		(0.002)	
Medical service satisfaction	-0.0003	-0.05	0.066	1.92
	(0.006)		(0.034)	
Number of hospitalizations in a year	-0.012	-1.81	-0.041	-0.91
	(0.007)		(0.046)	
Received medicaid last year	-9.61E-06^a^	-2.48	-0.0003^a^	-2.80
	(3.87E-06)		(0.0001)	
Constant term	9.218^a^	60.22	7.203^a^	14.47

^a^means significant at the 5% level, the brackets represent standard error

According to the above model, the vulnerability equation of health poverty can be obtained:

$$\mathrm{LnYh}+1=-0.032\times{\mathrm a}_1-0.071\times{\mathrm a}_2-0.124\times{\mathrm a}_4-0.005\times{\mathrm a}_5+0.033\times{\mathrm a}_{14}+0.123\times{\mathrm a}_{15}-0.484\times{\mathrm a}_7-0.053\times{\mathrm a}_{11}-9.61\mathrm E-06\times{\mathrm a}_{10}+9.218$$

In the formula, a1 stands for “Self-evaluation of health”; a2 indicates “Pain”; a4 means “Gender”; a5 means “Age”; a7 indicates “Participating in the new rural cooperative medical insurance”; a10 means “Received medicaid last year”; a11 means “Master self-treatment”; a14 means “Checking blood pressure regularly”; a15 means “Checking for diabetes often”.

We can see from the above equation of the coefficient of the independent variables that income per capita of the rural elderly and gender, pain, age, participating in the new rural cooperative medical insurance, mastering self-treatment and received medicaid last year were negatively correlated, and regular blood pressure and diabetes checking were positively correlated.

The health poverty vulnerability of the rural elderly population in 2018 is shown in Table 6. When the poverty line is 2995 CNY/year, the health poverty vulnerability of the rural elderly population in China is 0.397; that is, the probability of the rural elderly population falling into poverty due to health factors in 2018 is 39.7%. When the poverty line of 4589 CNY/year is used, our rural elderly population health poverty vulnerability is 0.598, which shows that the higher the poverty line is set, the greater the vulnerability is.

**Table 6:** Health poverty vulnerability of the elderly in rural China

The poverty line	2995 CNY/year		4589 CNY/year	
Vulnerability to health poverty	0.397		0.598	

### Quantile regression analysis

In this paper, the health poverty vulnerability of the elderly in rural China with poverty lines of 2995 CNY/year and 4589 CNY/year were used as dependent variables. Nine quantiles, 0.1, 0.2, 0.3, 0.4, 0.5, 0.6, 0.7, 0.8 and 0.9, were selected to conduct quantile regression. In addition, 0.1–0.3 was classified as low health poverty vulnerability, 0.4–0.6 as medium health poverty vulnerability, and 0.7–0.9 as high health poverty vulnerability to explore whether the independent variable informal social support (X1: Relationship with spouse; X2: Relationship with children; X3: Financial support from children; X4: Sibling support; X5: Support from other friends and relatives; X6: Borrowing costs) is the influencing factor of the health poverty vulnerability of the elderly in rural China at different levels of vulnerability.

### Significance of different quantiles based on the 2995 CNY/year poverty line

According to the data in Table 7, relationship with spouse, relationship with children, borrowing costs, support from other friends and relatives, and sibling support have a significant impact on the health poverty vulnerability of the rural elderly population. Among them, borrowing costs are significant at the t = 0.5, t = 0.6 and t = 0.7 levels in quantile regression. The variable of relationship with spouse was not significant at the level of t = 0.1, but it was significant at the other loci. The relationship with children was significant only at the level of t = 0.1, and no significant effect was found at the other loci, indicating that the relationship with children only affected elderly individuals with low vulnerability levels. Sibling support was significant at t = 0.7 but had no significant effect at other loci. Support from other relatives and friends had an effect at the t = 0.7 and t = 0.9 loci but had no significant effect at other loci.

**Table 7:** Quantile regression results of the impact of informal social support on the health poverty vulnerability of the elderly according to the 2995 CNY/year poverty line

Variables	t = 0.1	t = 0.2	t = 0.3	t = 0.4	t = 0.5	t = 0.6	t = 0.7	t = 0.8	t = 0.9
Relationship with spouse	0.007	0.011*	0.011*	0.012*	0.013*	0.013*	0.012*	0.008*	0.007*
	(0.234)	(0.000)	(0.000)	(0.000)	(0.000)	(0.000)	(0.000)	(0.000)	(0.000)
Relationship with Children	-0.025*	-0.005	-0.002	-0.001	-0.002	0.001	-0.001	0.001	0.001
	(0.020)	(0.067)	(0.247)	(0.519)	(0.376)	(0.902)	(0.400)	(0.855)	(0.503)
Financial support from children	-9.93e-08	-3.01e-07	-2.15e-07	-2.82e-07	-8.77e-08	-1.71e-07	-1.58e-07	-8.69e-08	-1.15e-07
	(0.890)	(0.354)	(0.393)	(0.223)	(0.547)	(0.262)	(0.292)	(0.230)	(0.541)
Sibling support	-0.001	-0.001	-0.001	-0.001	-0.001	-0.001	-0.001*	-0.001	-0.001
	(0.887)	(0.103)	(0.305)	(0.223)	(0.072)	(0.104)	(0.028)	(0.142)	(0.798)
Support from other friends and relatives	6.29e-07	-2.31e-07	-1.81e-07	-3.39e-07	-5.52e-07	-4.49e-07	-6.53e-07*	-4.52e-07	-5.28e-07*
	(0.691)	(0.582)	(0.594)	(0.300)	(0.051)	(0.108)	(0.033)	(0.198)	(0.043)
Borrowing costs	-7.50e-08	-4.35e-08	-3.87e-08	-5.25e-08	-6.62e-08*	-8.06e-08*	-8.08e-08*	-9.12e-08	-6.48e-08
	(0.608)	(0.768)	(0.633)	(0.325)	(0.029)	(0.006)	(0.028)	(0.105)	(0.107)
Constant term	(0.000)	(0.000)	(0.000)	(0.000)	(0.000)	(0.000)	(0.000)	(0.000)	(0.000)

* means significant at the 5% level, the brackets represent *P* value

### Different quantile regression coefficients based on the 2995 CNY/year poverty line standard

**Fig. 2:**
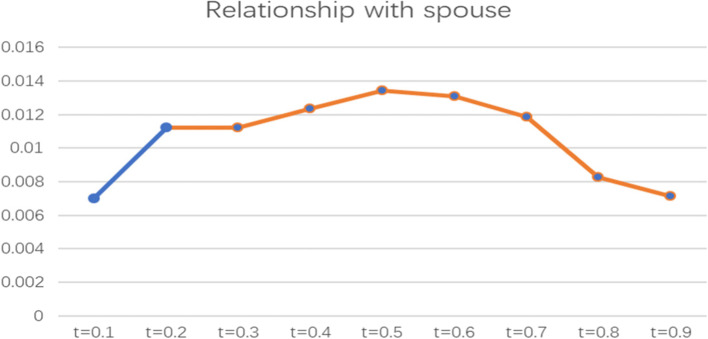
Line chart of the quantile regression coefficient of the relationship with spouse with a poverty standard of 2995 CNY/year

**Fig. 3:**
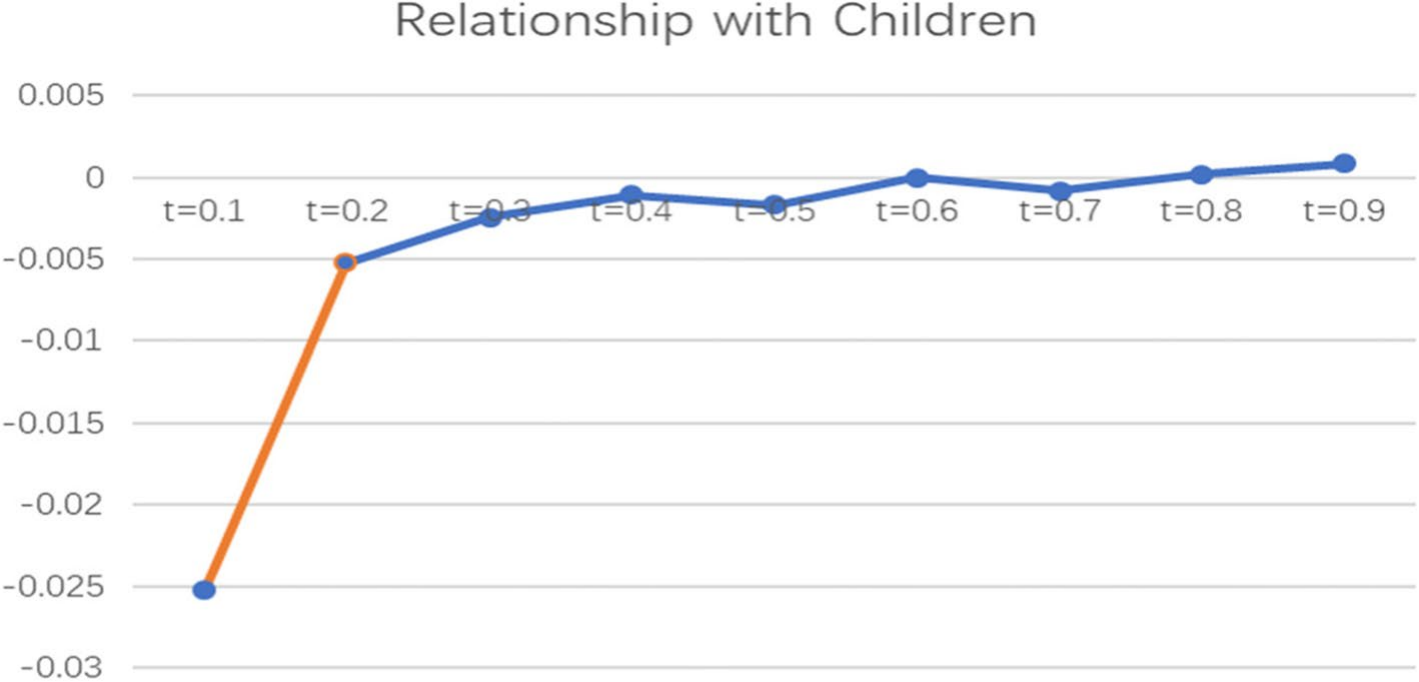
Line chart of the quantile regression coefficient of the relationship with children with a poverty standard of 2995 CNY/year

**Fig. 4:**
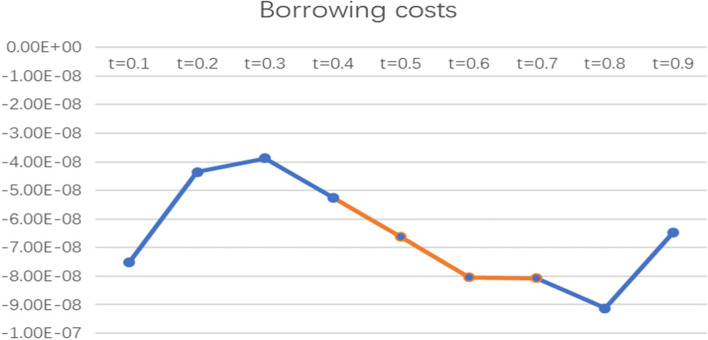
Line chart of the quantile regression coefficient of borrowing costs for the poverty standard of 2995 CNY/year

**Fig. 5:**
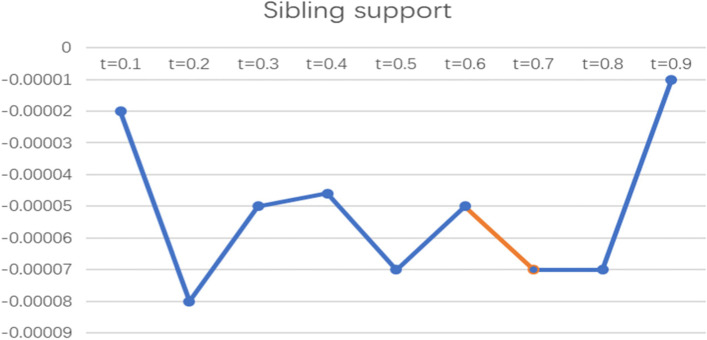
Line chart of the quantile regression coefficient of sibling support for the poverty standard of 2995 CNY/year

**Fig. 6:**
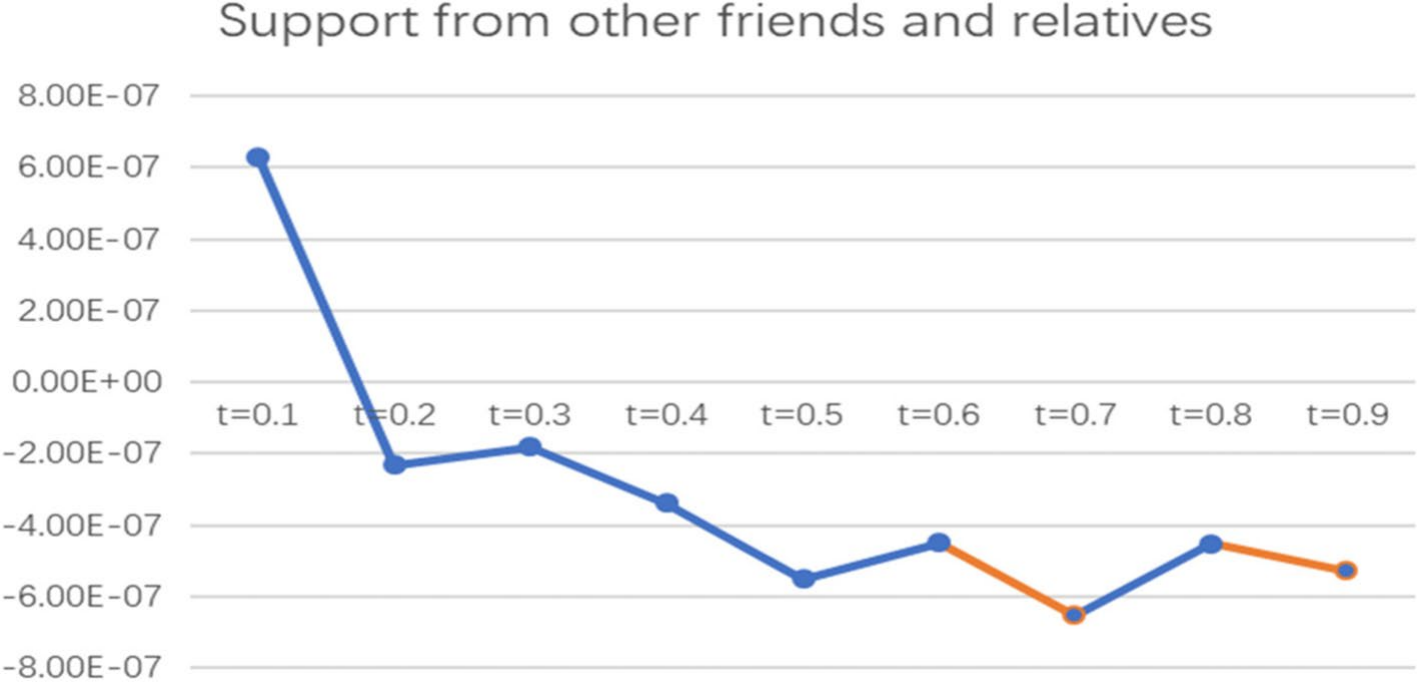
Line chart of the quantile regression coefficient of support from other friends and relatives for the poverty standard of 2995 CNY/year. Note: The orange segment is the indicator that is significant in this locus interval; the blue segment is not significant in this interval

To more intuitively observe the impact of informal social support factors on the health poverty vulnerability of the rural elderly in different quantiles of the poverty line of 2995 CNY/year, the line graph of the regression coefficients of various indicators of informal social support in different quantiles was drawn and analysed, as shown in Figs. 2, 3, 4, 5 and 6.

From the perspective of the relationship with spouse, the coefficient is above the X-axis, indicating that spousal relationships have a positive and significant impact on the health poverty vulnerability of the rural elderly population. The paper adopts the assignment method of the CHARLS Database, assigning “Extremely satisfied” as 1, “Greatly satisfied” as 2, “Satisfied” as 3, " Less satisfied” as 4, and “Completely dissatisfied” as 5; that is, the higher the value is, the lower the satisfaction. Thus, the spouse relationship has a positive effect, indicating that the higher the satisfaction of the spouse relationship of elderly individuals, the lower the health poverty vulnerability. This coefficient is the highest when t = 0.5 and the lowest when t = 0.9, showing an overall trend of increasing first and then decreasing. Moreover, this indicator has no impact on the elderly at low levels. However, it has a more significant impact on elderly people with medium and high levels of vulnerability.

The coefficient of the relationship with children indicates that this variable only affects elderly people with low vulnerability levels and has no significant effect on the health poverty vulnerability of elderly people with middle and high vulnerability levels. The coefficient fluctuates below the X-axis, indicating that satisfaction with the relationship between the elderly and their children has a negative impact on the low vulnerability groups; that is, the better the relationship with children is, the higher the vulnerability to health poverty of the rural elderly population.

From the coefficient of borrowing cost, the indicator has an impact at t = 0.5, t = 0.6 and t = 0.7 and shows a trend of fluctuation below the X-axis, indicating that borrowing costs have a negative impact on the health poverty vulnerability of the elderly at the middle level of vulnerability; that is, the lower the borrowing costs are, the higher the health poverty vulnerability of the elderly population.

In terms of sibling support, the coefficient was below the X-axis and only had a significant effect at t = 0.7, while the other sub loci had no effect. This indicates that this indicator has a negative effect on the elderly with a high level of vulnerability but has no significant effect on the elderly with low and medium levels of vulnerability, and the more sibling support there is, the lower the vulnerability to health poverty.

It can be seen from the coefficient diagram of support from other friends and relatives that the coefficient floats around the X-axis and is significant at t = 0.7 and t = 0.9, indicating that this indicator has a negative impact on elderly people with high levels of vulnerability but has no significant impact on elderly people with low and medium levels of vulnerability.

### Significance of different quartiles based on the 4589 CNY/year poverty line

According to the data in Table 8, the relationship with spouse, sibling support, support from other friends and relatives and borrowing costs have a significant impact on the health poverty vulnerability of the rural elderly population. Among them, the relationship with spouse was not significant at the level of t = 0.1 but was significant at the other sub loci. Sibling support was only significant at the level of t = 0.8 and had no significant effect at the other loci, indicating that sibling support has an impact on elderly people with high vulnerability levels. Support from friends and relatives was significant at the t = 0.9 level but had no significant effect at other loci. Borrowing costs are affected at t = 0.5, t = 0.6, and t = 0.7.

**Table 8:** Quantile regression results of the impact of informal social support on the health poverty vulnerability of the elderly according to the 4589 CNY/year poverty line

Variables	t = 0.1	t = 0.2	t = 0.3	t = 0.4	t = 0.5	t = 0.6	t = 0.7	t = 0.8	t = 0.9
Relationship with spouse	0.007	0.011*	0.010*	0.011*	0.012*	0.012*	0.010*	0.007*	0.006*
	(0.322)	(0.000)	(0.000)	(0.000)	(0.000)	(0.000)	(0.000)	(0.000)	(0.000)
Relationship with Children	-0.025	-0.005	-0.002	-0.001	-0.001	0.001	-0.001	0.001	0.001
	(0.090)	(0.107)	(0.401)	(0.581)	(0.513)	(0.805)	(0.574)	(0.835)	(0.385)
Financial support from children	-1.04e-07	-2.94e-07	-2.06e-07	-2.66e-07	-7.69e-08	-1.55e-07	-1.43e-07	-7.41e-08	-9.63e-08
	(0.896)	(0.311)	(0.248)	(0.243)	(0.536)	(0.261)	(0.298)	(0.401)	(0.317)
Sibling support	-0.001	-0.001	-0.001	-0.001	-0.001	-0.001	-0.001	-0.001*	-0.001
	(0.826)	(0.096)	(0.289)	(0.291)	(0.103)	(0.303)	(0.141)	(0.021)	(0.796)
Support from other friends and relatives	6.36e-07	-2.32e-07	-1.73e-07	-3.10e-07	-4.85e-07	-4.08e-07	-5.85e-07	-3.85e-07	-4.48e-07*
	(0.738)	(0.624)	(0.573)	(0.305)	(0.105)	(0.202)	(0.093)	(0.192)	(0.047)
Borrowing costs	-7.30e-08	-4.28e-08	-3.65e-08	-5.06e-08	-6.34e-08*	-7.64e-08*	-6.91e-08*	-8.03e-08	-5.51e-08
	(0.744)	(0.796)	(0.532)	(0.144)	(0.015)	(0.002)	(0.006)	(0.116)	(0.188)
Constant term	(0.000)	(0.000)	(0.000)	(0.000)	(0.000)	(0.000)	(0.000)	(0.000)	(0.000)

* means significant at the 5% level, the brackets represent *P* value

### Different quantile regression coefficients based on the 4589 CNY/year poverty line standard

**Fig. 7:**
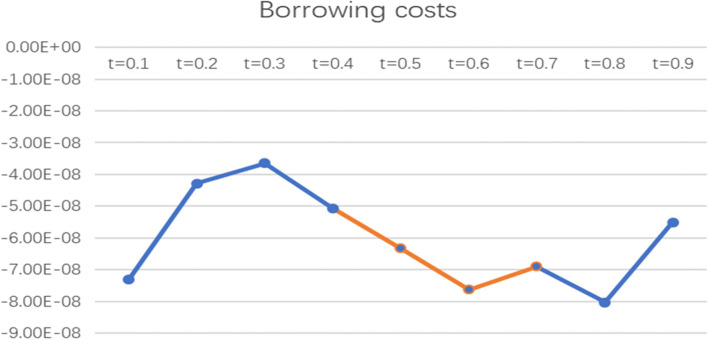
Line chart of the quantile regression coefficient of borrowing costs for the poverty standard of 4589 CNY/year

**Fig. 8:**
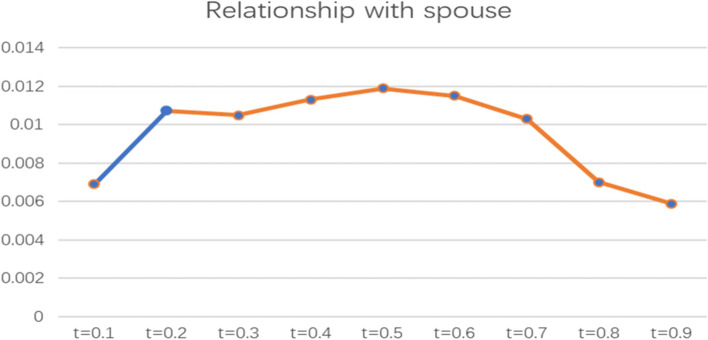
Line chart of the quantile regression coefficient of the relationship with spouse for the poverty standard of 4589 CNY/year

**Fig. 9:**
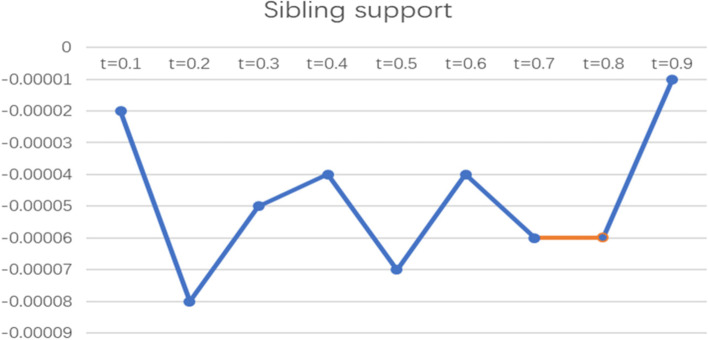
Line chart of the quantile regression coefficient of sibling support for the poverty standard of 4589 CNY/year

**Fig. 10:**
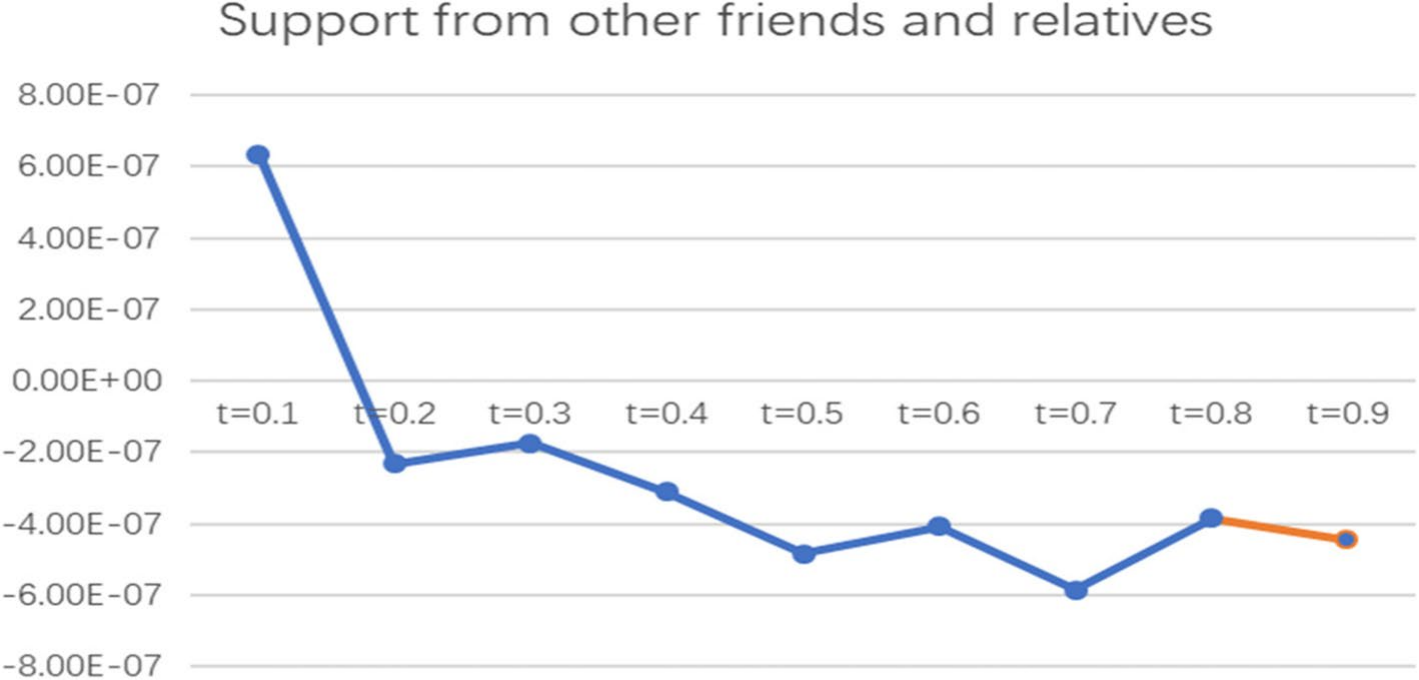
Line chart of the quantile regression coefficient of support from other friends and relatives for the poverty standard of 4589 CNY/year. Note: The orange segment is the indicator that is significant in this locus interval; the blue segment is not significant in this interval

To more intuitively observe the impact of social support factors on the health poverty vulnerability of the rural elderly in different quantiles of the poverty line of 4589 CNY/year, the line graph of regression coefficients of various indicators of social support in different quantiles was drawn and analysed, as shown in Figs. 7, 8, 9, and 10.

From the perspective of the relationship with spouse, the coefficient is above the X-axis, indicating that spousal relationships have a positive and significant impact on the health poverty vulnerability of the rural elderly population. The paper adopts the assignment method of the CHARLS Database, assigning “Extremely satisfied” as 1, “Greatly satisfied” as 2, “Satisfied” as 3, " Less satisfied” as 4, and “Completely dissatisfied” as 5; that is, the higher the value is, the lower the satisfaction. Thus, the spouse relationship has a positive effect, indicating that the higher the satisfaction of the spouse relationship of elderly individuals, the lower the health poverty vulnerability. This coefficient was the highest when t = 0.5 and the lowest when t = 0.9 and showed a trend of slow rise and then decline as a whole. Moreover, this indicator has a more significant impact on the elderly with medium and high vulnerability levels.

The coefficient chart of sibling support shows that the indicator is significant at t = 0.8 and fluctuates below the X-axis, indicating that sibling support has a negative impact on the health poverty vulnerability of the elderly at the high score locus; that is, the less sibling support there is, the higher the health poverty vulnerability of the elderly population.

For support from other friends and relatives, the coefficient fluctuates along the X-axis and only has a significant effect at the level of t = 0.9, while the other subloci have no effect. This indicates that this indicator has a negative impact on the elderly with a high level of vulnerability but has no significant effect on the elderly with low and medium levels of vulnerability; that is, the more support from other friends and relatives there is, the lower the vulnerability to health poverty.

As shown from the coefficient chart of borrowing costs, the coefficient is below the X-axis and has a significant effect when t = 0.5, t = 0.6 and t = 0.7, indicating that this indicator has a negative impact on elderly people with medium-high levels of vulnerability but has no significant impact on elderly people with a low level of vulnerability.

## Discussion

The empirical results of this paper show that the relationship with spouse has a significant positive impact on the health poverty vulnerability of elderly individuals; the higher the satisfaction of the spouse relationship is, the lower the health poverty vulnerability of elderly individuals, and this effect is more significant in the elderly with high vulnerability. This is consistent with the previous research conclusions of scholars. Qin Xu proposed that informal social support is characterized by uncertainty, which is manifested by the relationship between individuals [38]. Yuan Yao believes that interpersonal relationships and emotional principles drive the operation of informal social support [39]. Sociological and psychological studies show that marital status is one of the important factors affecting the physical and mental health of the elderly [40]. The spousal relationship can meet the emotional needs of elderly individuals, improve their confidence, help to enhance the communication between the elderly and society, and encourage them to realize their own value, which is consistent with the previous research conclusions. The reason is that rural households and their families have been living in rural areas for a long time, and the relationship between their spouses and action subjects is relatively close. Elderly individuals are in a poverty trap when their health is poor, but their spouses can play a greater role in daily care. A good relationship between the elderly and their spouses can alleviate their anxiety and other negative emotions caused by the lack of spiritual support, reducing the possibility of their psychological causes of disease and ultimately reducing their health poverty vulnerability.

The empirical results of this study show that borrowing costs have a significant negative impact on elderly people with moderate vulnerability; that is, for elderly people with moderate vulnerability, the greater the borrowing costs are, the lower the vulnerability to health poverty. This result is also consistent with the conclusions of other scholars. Adams R showed that unofficial social assistance, such as support provided by neighbors, mainly includes loans, transportation and other practical help [21]. Yuan Yao pointed out that for the elderly in rural areas, unofficial social assistance from families, neighbors and charity organizations can meet their needs [41]. The help provided by nonrelatives such as neighbours is different from the financial support provided by children. When emergencies occur, the support from neighbors is the most direct and immediate [42]. This paper argues that the elderly population with moderate vulnerability is at the edge of poverty due to low income, poor health and other reasons. Therefore, more borrowing costs are incurred. With economic security, the probability of falling into the poverty trap decreases in the future. However, the impact of borrowing costs on the elderly with low or high vulnerability levels is not significant. The analysis is based on the following reasons: on the one hand, the economic burden of medical expenses makes the elderly borrow through unofficial channels, which cannot fundamentally solve the actual situation of them. On the other hand, the elderly with a high level of vulnerability or even the whole family lack the ability of loan repayment and need the government to provide them with more accurate assistance. Therefore, this indicator of borrowing costs has no significant impact on them.

According to the poverty criteria of 2995 CNY/year and 4589 CNY/year, sibling support and support from other friends and relatives have a significant negative impact on the health poverty vulnerability of the elderly at the high score locus; that is, the more sibling support and support from other friends and relatives there are, the lower the health poverty vulnerability of the elderly is, and has nonsignificant effect on the middle and low levels of vulnerability. This conclusion shows that support from other friends and relatives can alleviate poverty to some extent for elderly people with high locus points and reduce the possibility of falling into the health poverty trap. This empirical conclusion is consistent with the views of previous scholars. Zhenyu Su pointed out that support from relatives and friends is an important part of the informal social support system for the elderly [43]. Dongfang Li believed that informal social support enhances rural residents’ access to medical services and enhances the sharing of disease risk among relatives and friends, which can significantly promote the health status of rural residents [44]. Xiaoliang Hong and Zhigang Yin believed that families with high poverty vulnerability can also seek help and support from close relatives, including spiritual and economic support, while making efforts by themselves, and economic support from close relatives can play a significant role [24]. Gang Li found that relative relationships play a greater role in supporting poor families than nonrelative relationships, and relatives can provide labor support and free economic support for poor families [31]. Sherraden found that family support can help poor women obtain more supplies in terms of nutrition, housing and physical health, moderating the impact of moderate poverty [45]. Min Li believed that when formal institutional support is unequal and scarce, informal social support in the private sector can help them effectively cope with economic difficulties [46]. Jun Tang’s case study of poor families in Shanghai shows that the most important support for poor families comes from relatives, including education expenses, medical expenses, daily living expenses, clothing and other financial and emotional support [47].

Data analysis shows that the relationship with children only has a significant negative effect on the health poverty vulnerability of the rural elderly with low vulnerability; that is, the better the relationship with children is, the higher the health poverty vulnerability of elderly individuals. This conclusion is quite different from the previous research conclusions of scholars. Zhaiping He believed that informal social support is a hierarchical network, with the parent‒child relationship at the core, which is the most dynamic and most secure supporting factor in informal social support [48], while other relationships are located at different levels around this core. Yuan Yao pointed out that family support can provide spiritual and emotional comfort for the elderly and meet their economic and care needs [49]. In terms of daily care, the “troika” composed of spouse, son and daughter is the main body of family care [50], which meets the most important and deficient needs of the elderly and is an important force for internal poverty alleviation. However, the empirical results of this paper show that the better the relationship between the rural elderly and their children is, the higher their vulnerability to health and poverty. It may in fact that the rural elderly and their families live in rural areas for years, a close relationship between children and the elderly exists. However, the whole family is in a state of lack of technology and labor, mainly manifest as the working-age members in the family are unable to be employed or lack basic skills for employment due to a low educational level, etc. Ying Zhao believed that poverty is mainly caused by complex reasons, showing the characteristics of “multiple superposition”, such as the lack of labor ability of the young in age to take care of elderly individuals, the lack of ability to care for severely disabled by the mildly disabled, etc. [51]. Meanwhile, this indicator of the relationship between the elderly and their children has no significant impact on the elderly with medium-high vulnerability, which is caused by the fact that the elderly with medium-high vulnerability suffer from complex diseases for years and the care from their children cannot significantly improve their own health level.

### Limitations

In this paper, the vulnerability to health poverty and informal social support of the rural elderly population in different regions of China were taken as dependent and independent variables, so the sample size was relatively small. As a prospective monitoring index, health poverty vulnerability is transformed from postmeasurement to premeasurement, which can make up for the shortcomings of traditional poverty measurement indexes. However, we measure poverty only in terms of a single dimension of income. In future studies, we will try to select other variables and expand the sample size to make up for the shortcomings of existing studies.

## Conclusion

The empirical results show that informal social support has a significant impact on the health poverty vulnerability of the rural elderly. Specifically, the higher the degree of relationship satisfaction with the spouse is, the lower the health poverty vulnerability of the rural elderly. Meanwhile, in the middle- and high-vulnerability elderly group, the more sibling support there is, the more support from other friends and relatives, and the more borrowing costs there are, the lower the vulnerability to health poverty, but the impact on the low-vulnerability elderly group is not significant. In addition, the relationship between the rural elderly and their children only significantly affects the probability of returning to poverty among the elderly population with low vulnerability.

Therefore, to reduce the vulnerability of the rural elderly to health poverty, we can consider the factor of informal social support. The country should fully implement the rural revitalization strategy and provide more job opportunities to attract migrant workers to return to their hometowns to work to jointly build villages with thriving industries and prosperous lives and shorten the space between empty nesters in rural areas and their children. For elderly people with a high level of vulnerability, the impact of family support is limited, and it is necessary to pay attention to the government, nongovernmental organizations and other channels of support. On the one hand, administrative organizations dominated by the government are the backbone of health poverty alleviation work and should give full play to their roles in diagnosis and treatment, health education, basic medical insurance and minimum living security [52]. On the other hand, in addition to the social support provided by the government, nongovernmental organizations should also play an important part in complementing the government’s work in the form of the provision of funds, participation in social assistance and emotional support.

## Data Availability

The database (CHARLS2018), which is used in this paper, is publicly available. (http://charls.pku.edu.cn/en).

## Abbreviations

CHARLS: China Health and Retirement Longitudinal Study
FGLS: Feasible Generalized Least Squares
